# Increased Thyroid Cancer Incidence in Volcanic Areas: A Role of Increased Heavy Metals in the Environment?

**DOI:** 10.3390/ijms21103425

**Published:** 2020-05-12

**Authors:** Pasqualino Malandrino, Marco Russo, Fiorenza Gianì, Gabriella Pellegriti, Paolo Vigneri, Antonino Belfiore, Enrico Rizzarelli, Riccardo Vigneri

**Affiliations:** 1 Endocrinology, Department of Clinical and Experimental Medicine, University of Catania, Garibaldi-Nesima Medical Center, 95122 Catania, Italy; p.malandrino@unict.it (P.M.); mruss@hotmail.it (M.R.); fiorenza.giani@gmail.com (F.G.); g.pellegriti@unict.it (G.P.); antonino.belfiore@unict.it (A.B.); 2 Medical Oncology and the Center of Experimental Oncology and Hematology, Department of Clinical and Experimental Medicine, University of Catania, A.O.U. Policlinico Vittorio Emanuele, 95125 Catania, Italy; pvigneri@libero.it; 3 Department of Chemical Sciences, University of Catania, 95125 Catania, Italy; erizzarelli@unict.it; 4 Consiglio Nazionale delle Ricerche, Cristallography Institute (Catania Section), via P. Gaifami 18, 95126 Catania, Italy; 5 Consorzio Interuniversitario di Ricerca in Chimica dei Metalli nei Sistemi Biologici (CIRCMSB), via Celso Ulpiani 27, 70126 Bari, Italy

**Keywords:** thyroid, thyroid cancer, volcano, metals, metallome, carcinogens, hormesis, environment pollution, metal biocontamination

## Abstract

Thyroid cancer incidence is significantly increased in volcanic areas, where relevant non-anthropogenic pollution with heavy metals is present in the environment. This review will discuss whether chronic lifelong exposure to slightly increased levels of metals can contribute to the increase in thyroid cancer in the residents of a volcanic area. The influence of metals on living cells depends on the physicochemical properties of the metals and their interaction with the target cell metallostasis network, which includes transporters, intracellular binding proteins, and metal-responsive elements. Very little is known about the carcinogenic potential of slightly increased metal levels on the thyroid, which might be more sensitive to mutagenic damage because of its unique biology related to iodine, which is a very reactive and strongly oxidizing agent. Different mechanisms could explain the specific carcinogenic effect of borderline/high environmental levels of metals on the thyroid, including (a) hormesis, the nonlinear response to chemicals causing important biological effects at low concentrations; (b) metal accumulation in the thyroid relative to other tissues; and (c) the specific effects of a mixture of different metals. Recent evidence related to all of these mechanisms is now available, and the data are compatible with a cause–effect relationship between increased metal levels in the environment and an increase in thyroid cancer incidence.

## 1. Introduction

Thyroid cancer is the most frequent endocrine cancer (1.0%–1.5% of all new cases in the US), and its incidence, which was stable until the 1980s, has constantly increased since that time [1,2]. It is now the fourth most frequent cancer in women [3], whereas it was the 14th in the early 1990s.

This increase has occurred worldwide, as documented by the annual percent change in all countries where this parameter has been calculated with very few exceptions [4,5].

The reasons underlying these changes are unknown. Many experts believe that the apparent increase is mostly due to the overdiagnosis of small papillary thyroid tumors without significant clinical relevance that were not detected in the past but have been identified in the last decades because of the increasing diffusion of sensitive imaging procedures such as ultrasound scans [6]. Although there is a general consensus that a higher detection rate contributes to the increasing thyroid cancer incidence, much evidence indicates that this cannot be the only explanation. In fact, large thyroid cancers have also increased [7], and these tumors are very unlikely to have gone undetected in the past. Moreover, thyroid cancer-related mortality, which should have decreased because of early detection and better treatment, is stable or increasing [8]. Finally, a temporal trend in the changes in the thyroid cancer molecular profile characterized by an increasing prevalence of BRAF and RAS mutations [2,9,10] supports the possibility that a true change in thyroid cancer biology is occurring.

The causes of these recent changes in both quantitative and qualitative thyroid cancer characteristics are most likely environmental, as suggested by the sharp increase in incidence during the last decades. Most malignancies have not increased within this period, indicating that the potential carcinogenic factors involved must in some way be thyroid specific. Therefore, general factors that are known to favor cancer, such as the obesity epidemic, are unlikely to play a major role specifically in thyroid cancer.

Other more plausible environmental risk factors have been suggested. Since the thyroid is very radiosensitive, especially at a young age, and exposure to radiation has doubled in the last 25 years in most industrialized countries (mainly because of medical diagnostic procedures), radiation is the most commonly referenced cause [11,12]. The higher radiation-related carcinogenicity in the thyroid might be a consequence of frequent dental X-rays in children/adolescents.

Another possible risk factor specific to the thyroid is the progressive increase in iodine intake due to prophylaxis programs for iodine deficiency and goiter carried out worldwide in recent decades. Iodine enrichment may favor chronic lymphocytic thyroiditis [13], which may in turn promote thyroid cancer by increasing thyroid-stimulating hormone (TSH) levels and inducing proinflammatory cytokine production and oxidative stress in the gland [14,15,16].

However, the large number of potential carcinogens associated with the westernized postindustrial lifestyle should also be considered. In recent decades, the population has been highly and progressively exposed to compounds and chemicals that may interfere with biological functions, including hormone homeostasis (endocrine disruptor chemicals) [17]. Many compounds used in agroindustrial activities (fertilizers, pesticides, repellents, and preservatives) may directly cause cancer or indirectly produce conditions that favor malignant transformation. For instance, the increased ingestion of nitrates, which are a frequent contaminant of drinking water in areas of intense agricultural industry and are present at high levels in processed meat, has been associated with an increased risk of thyroid cancer [18,19]. Other potential thyroid-specific carcinogens can originate from other industrial activities such as the organic compounds polybrominated diphenyl ethers and bisphenols [20,21,22]. However, many other environmental pollutants (solvents, plastic, heavy metals, diet preservatives, etc.) may be responsible for the increased thyroid cancer. Therefore, further investigations are warranted to identify these potential carcinogens and their mechanism of action on the thyroid to introduce preventive measures aimed at controlling the continuous thyroid cancer increase.

## 2. Volcanic Environment and Thyroid Cancer

A specific natural example of the thyroid cancer–environment relationship is the increased incidence of this cancer observed in residents of volcanic areas.

This association was first reported 40 years ago [23] and was then confirmed by observations made on islands with active volcanoes, such as Hawaii [24,25] and Iceland [26,27], in the late 1980s. An elevated thyroid cancer incidence has since been reported in numerous volcanic areas in the Pacific Ocean, such as Vanuatu [28], French Polynesia [29], and New Caledonia [30], leading to the hypothesis that several components of volcanic lava could be involved in the pathogenesis of thyroid cancer [31].

Many possible causative factors have been proposed, including the possibility that genetic characteristics present on isolated islands with a small population might be responsible. However, the observation that residents of Hawaii exhibit much higher rates of thyroid cancer relative to individuals with the same ethnic background living in other geographic areas suggests that environmental rather than genetic influences play a major role [24]. Among these environmental factors, geothermal causes such as high-temperature water containing hydrogen sulfide and radon [32] have been hypothesized to play a role, as has the possibility that specific dietary factors favoring thyroid cancer exist in these areas. The increased natural radioactivity found in the volcanic areas and mainly due to ^222^Radon emission might also play a role, but recent studies found no association between Radon levels and thyroid cancer [33,34].

In the early 2000s, an epidemiological study was carried out in Sicily, which is a large Mediterranean island with over five million inhabitants on which a continuously active volcano (Mt. Etna, the highest volcano in Europe) is located in the northeastern area in the province of Catania. This volcanic area has a population of over 1,000,000 inhabitants, and it was therefore possible to compare two large populations with the same ethnic background, similar sex and age distributions, similar lifestyles, and similar access to medical assistance.

The incidence of thyroid cancer was more than doubled in residents of the volcanic area compared to the remaining population of Sicily [35]. The F/M ratio was 4.8:1, with no significant difference between the two areas. An increased incidence was also present in pediatric age [36]. Environmental factors such as iodine intake and industrial pollution did not differ in the two areas. Moreover, only thyroid cancer of the papillary histotype was increased in the volcanic area, reflecting the observation that this histotype is the main cause of the worldwide increase in thyroid cancer incidence (Table 1).

Therefore, the findings in the Mt. Etna volcanic area strongly suggest an association between the volcanic environment and the increase in thyroid cancer with a cause–effect relationship but with no indication of the possible causative factors and their mechanisms of action.

Active volcanoes cause considerable non-anthropogenic pollution due to gas, ash, and lava emissions. This pollution may have different characteristics among different volcanoes depending on the chemical, physical, and geologic characteristics of each volcano and its effusive activity. In all cases, a variety of elements originating from the depths under the Earth’s crust pollute the atmosphere, water, soil, and food and will cause biocontamination in the resident population via these routes [37,38]. Within these forms of pollution, heavy metals may play an important role. These natural components of the Earth’s soil show complex interactions with organic elements (e.g., amino acids, carbohydrates, and nucleotides) and play an important role in biological events, including those related to cell growth and transformation [39].

## 3. Biological Bases of Metal Homeostasis

Once metals from the Earth’s crust become available in the environment, they cannot be degraded or destroyed and play an important role in the biology of plants and animals. Some metals that are essential nutrients in trace amounts may become toxic compounds at higher concentrations. Other metals are toxic and carcinogenic even at very low levels. Therefore, the homeostatic regulation of metals is under strict control in cells, including the sensing, transport, and accumulation of metals. However, the underlying regulatory mechanisms are poorly understood: metal-dependent processes may influence many aspects and functions of cell biology via mechanisms (and at concentrations) that are still unclear for many metals in many instances. Nature employs the unique chemical features of essential micronutrients belonging to the block d metals, such as Zn (Zinc), Cu (Copper), Ni (Nickel), and Co (Cobalt), because of their donor atom preferences, coordination structures, and redox capacity. These characteristics are important to obtain a reliable repertoire of structural and catalytic functions of many proteins.

Many of these proteins are present at high concentrations, and significant amounts of the associated metal ions are required to guarantee their functions. Tightly bound metal pools buried within proteins are found in the cytosol as well as all cell organelles, including the endoplasmic reticulum, Golgi, mitochondria, lysosomes/vacuoles, and nucleus. Metals in this form significantly contribute to the total metal ion content (0.01–1 mM) needed for optimal living cell survival [40,41].

In sharp contrast to the high level of metals bound to proteins (*static metallome*), the concentration of block d metal ions that are not tightly bound to proteins (*dynamic metallome*, i.e., labile or exchangeable metal ions) is very low. The analysis of metal-responsive sensor molecules [42] indicated that the level of labile cytosolic metal ions is in the pM–nM range, i.e., 5–9 orders of magnitude lower than that of the static metallome pool [43,44]. These findings demonstrate the need for an efficient network of players in metallostasis (metal homeostasis) that can provide a sufficient supply of metal ions for protein function while maintaining the dynamic metallome at an extremely low level [45,46]. This network can ensure metal homeostasis within cells even when extracellular levels of metal ions vary over a large range.

The main players in the metallostasis network are solute carriers (SLCs), which play a vital role in the healthy functioning of living cells, exerting strict control over the import and export of ions, metabolites, and nutrients across membranes [47]. It has been estimated that approximately 10% of the human genome is linked to the control of membrane transport [47]. The network also includes chaperones and storage molecules such as metallothionines (MTs) [48,49], transcription factors (TFs) [50,51] and small molecules involved in the detoxification system, such as glutathione (GSH) [52].

**Figure 1:**
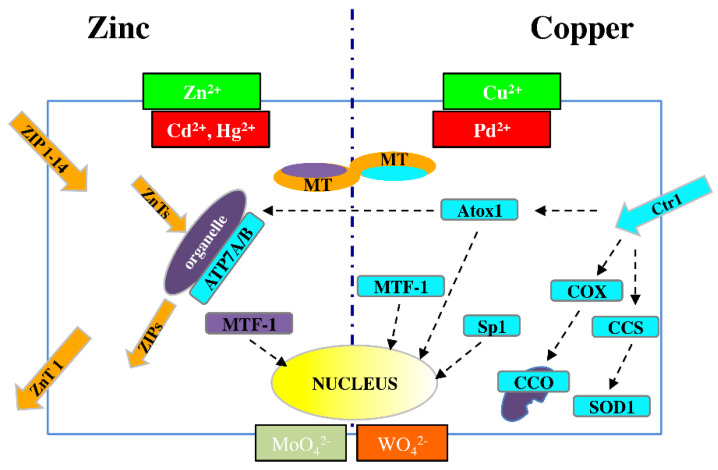
Schematic picture of dynamic metal homeostasis (metallostasis) players for Zn^2+^ (**left**) and Cu^2+^ (**right**). As a result of chemical similarities, Cd^2+^ and Hg^2+^ can compete with Zn^2+^, while Pd^2+^ can compete with Cu^+^ for cellular transport and binding proteins. Similar competition can occur between molybdenum and tungsten. ZIP (14 members) = membrane protein transporters for Zn^2+^ influx; ZnT (10 members) = membrane protein transporters for Zn^2+^ efflux; MT = metallothioneins; MTF-1 = metal-responsive transcription factor-1; APT7A/B = metal-transporting P-type ATPase A and B; Ctr1 = membrane protein for copper uptake; Atox1 = antioxidant 1 copper chaperon; COX = cytochrome c oxidase copper chaperon; CCO = cytochrome c oxidase; CCS = copper chaperon for superoxide dismutase; SOD1 = superoxide dismutase 1; Sp1 = transcription factor specific protein 1.

This fine control of the metallome is exemplified by the biology of the two best-characterized metal ions, Zn and Cu, both of which act as intracellular regulators of major signaling pathways [53] (Figure 1).

Zn transporters are classified into the two major families: SLC39A/ZIP (14 members) and SLC30A/ZnT (10 members), which are responsible for metal influx (both from outside the cell and from organelles to cytosol) and efflux (from cytosol to both outside the cell and to organelles), respectively [54,55]. The ZnT family includes eight cloned members, referred to as ZnT1-8 [54], and two others, ZnT9 and ZnT10, predicted from mouse and human genome resources [56].

A central role in metal homeostasis/detoxification is performed by metal-responsive transcription factor-1 (MTF-1), which binds DNA to modulate RNA transcription in response to altered cytosolic levels of Zn and/or Cu ions [57]; MTF-1 regulates the expression of both ZnT1 and MTs, which are proteins involved in metal release and storage, respectively [58].

For Cu, the high-affinity membrane copper transporter 1 (Ctr1) is the major cellular protein responsible for the uptake of this metal [59]. Minor SLCs for Cu include divalent metal ion transporter 1 (DMT1) and copper transporter 2 (Ctr2) [60].

Once the metal is inside the cell, intracellular chaperones transfer copper ions to specific targets. Such transfer is observed between copper chaperone for superoxide dismutase (CCS) and superoxide dismutase 1 (SOD1) in the cytosol; cytochrome c oxidase copper chaperone 17 (COX17) and cytochrome c oxidase (CCO) in mitochondria; and antioxidant 1 copper chaperone (Atox1) and ATPases such as copper-transporting P-type ATPase A and B (APT7A and APT7B) in the trans-Golgi network (TGN) [61,62,63].

When the copper concentration is excessive, Ctr1 is downregulated by specific protein 1 (Sp1), [64], which is a transcription factor that controls the homeostatic maintenance of Ctr1 mRNA. When there is a deficiency of these metal ions, Sp1 activity is reduced, and Ctr1 expression increases [55].

In addition, cytochrome c oxidase copper chaperone 11 (COX11) mRNA levels are related to Ctr1 expression and are upregulated in proliferating cells, in which there is the functional hyperactivity of copper trafficking pathways.

Owing to their chemical analogy to Zn^2+^, Cd^2+^ and Hg^2+^ may exert a competitive effect in binding to transporters and other coplayers in zinc homeostasis. The same is true for Pd^2+^ with respect to Cu^2+^, and similar competition may occur between Mo and toxic W (in the chemical forms of MoO_4_^2−^ and WO_4_^2−^)

For most other metals, similar complex machinery for extra- and intra-cellular metal trafficking might be present, but very little is known about their existence and function.

## 4. Metal Carcinogenicity and the Thyroid

Metals include both essential metals that are required micronutrients for biological processes (Fe: Iron, Zn, Cu, Se: Selenium, etc.) and toxic chemicals that may damage cell biology and promote malignant transformation (As: Arsenic, Cd: Cadmium, Hg: Mercury, Ni, etc.).

As a result of the numerous complex factors involved in the interaction of metals with living cells and the possible combined action of different mechanisms, our present understanding of the carcinogenic potential of a single metal or a mixture of different metals in living cells in general and the thyroid in particular is very limited.

The carcinogenic effect of metals on target cells depends on several biological factors, such as bioavailability (entering cells through the cell membrane), the intracellular distribution, and interactions with cellular proteins and enzymes. These steps are tissue and cell-specific.

Once a metal enters cells, its genotoxicity is generally exerted by indirect rather than direct actions on DNA. The most common mechanisms include (a) the induction of oxidative stress, which may in turn activate intracellular signaling leading to oxidative DNA damage; (b) interference with DNA repair systems, causing the accumulation of mutations; (c) the deregulation of growth control by damaging the balance between proliferative and apoptotic pathways; and (d) modification of the DNA methylation pattern, affecting the expression of oncogenes and oncosuppressors.

In relation to these mechanisms, an important factor is metal speciation (both inorganic and metal–organic), which determines the physicochemical properties and bioavailability of metals and, therefore, their biological effects.

For instance, high levels of As in drinking water, soil, food, and the atmosphere are associated with several types of cancer (As is a recognized human carcinogen belonging to group 1 according to International Agency for Research on Cancer-IARC classification), but the underlying mechanisms are not fully understood and are probably different for different As compounds.

In humans, As is relatively nontoxic when it occurs as a metallo-organic species in seafood (arsenobetaine) [65]. Among the inorganic forms of As, arsenate poses higher toxicity to endocrine glands than arsenite [66]. Moreover, a methylated arsenic compound (dimethyl arsenic acid) promotes carcinogenesis in many rat organs, including the thyroid [67], but another As derivative (arsenic trioxide) reduces proliferation and increases apoptosis and iodine uptake in papillary and follicular thyroid cancer cells [68], acting as a differentiating anticancer agent.

Additional mechanisms of As carcinogenesis may involve microRNA dysregulation. In arsenic-transformed human lung epithelial cells, miR-222 is upregulated, and its inhibition decreases cell proliferation and migration and increases apoptosis [69]. Notably, miR-222 may be significantly upregulated in papillary thyroid cancer [70]. Finally, in French Polynesia, an area with a high incidence of thyroid cancer, the risk of this cancer is increased by 30% for each increase in As intake of 1 µg/d/kg body weight, despite being within the recommended daily intake indicated by the WHO. However, this increase in thyroid cancer particularly affected individuals with first-degree relatives with a history of cancer [71], suggesting that As may act as a cocarcinogen with a combined effect with genetic susceptibility.

Cadmium is another carcinogenic metal of the IARC group This metal has been identified as an endocrine-disrupting chemical for many endocrine glands, including the thyroid [72], but it is also a carcinogen with multifactorial mechanisms. The physiochemical properties of Cd^2+^ ions allow them to substitute for calcium ions in biological systems because of showing the same charge and a similar radius and to use zinc transporters and substitute for Zn^2+^ in many enzymes and transcription factors, competing for Zn finger motifs [73,74,75].

Cd can induce oxidative stress by inhibiting antioxidant enzymes, activating the PI3K (phosphoinositide 3-kinase) and ERK (extracellular signal-regulated kinase) signaling pathways, deregulating cell proliferation, and damaging DNA repair mechanisms. Through one or more of these mechanisms, Cd can induce cancer initiation and progression [76]. An additional cancer-promoting effect of Cd is its disrupting effect on E-cadherin: by displacing Ca^2+^ from this protein, Cd disrupts cadherin-mediated cell–cell adhesion and therefore favors tumor progression and invasiveness [77].

Finally, Cd may favor thyroid cancer with a unique mechanism due to its metalloestrogen characteristics. Cd can in fact mimic the effects of 17B-estradiol on the G protein-coupled estrogen receptor. This receptor is present in thyroid follicular cells, and via its stimulation, Cd can promote the proliferation, invasion, and migration of thyroid cancer cells [78].

Many other metals exhibit carcinogenic activity, but most of them have never been directly tested in the thyroid. In any case, the available data are fragmentary, inconclusive, and sometimes contradictory. Moreover, in most of the relevant studies, only high metal concentrations and short-term effects were investigated, representing quite different conditions from the chronic exposure and the low-level metal increases generally found in association with environmental pollution.

## 5. Heavy Metals in the Mt. Etna Volcanic Area and Resident Biocontamination

Based on the observation that an increased trace element concentration may be present in volcanic areas [79,80] and, more specifically, that increased levels of metals such as B (Boron), Fe, Mn (Manganese), and V (Vanadium) are found in the groundwater of the Mt. Etna volcanic area [35], a careful comparative study of heavy metal environmental pollution and the biocontamination of residents was carried out in volcanic and control areas of Sicily [81].

To investigate environmental pollution, metal concentrations were measured in water and lichens. Mt. Etna harbors a large aquifer that provides water to over 700,000 residents and is used for irrigation in most of Catania Province. Therefore, volcanic aquifer-originating water is an important vehicle for population biocontamination, both directly and indirectly via locally grown food. Lichens are composite organisms that bioaccumulate elements present in the atmosphere and are therefore used for the biomonitoring of atmospheric pollution [82,83].

To investigate human biocontamination, urine specimens were collected from two matched groups of individuals living in volcanic and control areas. In fact, under conditions of chronic exposure, urine is considered a reliable indicator of the chemicals absorbed by a subject through contact, inhalation, and ingestion.

By measuring 27 trace elements and heavy metals in the environment (water and lichens) and human biological samples (urine), considerable volcanically derived biocontamination was found. In the volcanic area, where the thyroid cancer incidence is doubled, many metals were significantly increased in both water and lichens, documenting metal pollution in the environment. The differences relative to the control areas were more marked in water, in which the concentrations of metals such as As, B, Cd, Hg, Mn, Mo (Molybdenum), Pd (Palladium), Se, U (Uranium), V, and W (Tungsten) were increased by three- to 50-fold, although their average concentrations never exceeded the reference values indicated by the World Health Organization [84].

When these elements were measured in the urine specimens, 18 elements were found to occur at a significantly increased level compared to values measured in the urine samples collected in the control areas.

**Figure 2:**
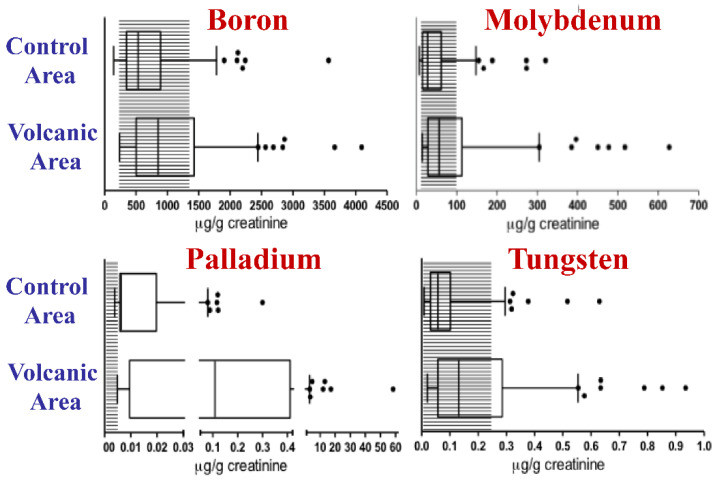
Metal biocontamination in the urine of residents of the volcanic area. The concentrations of boron, molybdenum, palladium, and tungsten were measured in the urine of 140 residents of the volcanic area and of 138 residents of the control area in Sicily. Data obtained from [81]. For B, Mo, Pd and W concentrations were significantly higher in the urine of residents of the volcanic area than in that of residents of the control nonvolcanic area and exceeded the normal reference values in over 20% of cases. The boxes indicate the 25th, 50th (median), and 75th percentiles. The whiskers indicate the 5th and 95th percentiles. The shaded area indicates the Italian reference values for urine. The dots indicate individuals with urinary concentrations higher than the 95th percentile.

In particular, the geometric mean value was two-fold or more than two-fold higher for eight metals: Cd, Hg, Mn, Pd, Tl, U, V, and W. Moreover, the values of B, Mo, Pd and W were higher than the 95th percentile of the Italian reference values in more than 20% of the urine specimens from the volcanic area [81] (Figure 2). This human biocontamination was confirmed by the increased concentration of metals in the scalp hair of children living in the Mt. Etna volcanic area [85].

These data document relevant metal pollution and consequent human biocontamination in subjects living in a volcanic area where the thyroid cancer incidence is greatly increased. The well-established carcinogenic effect of some metals and the observation that individuals living in volcanically active areas exhibit DNA damage more frequently than subjects living in nonvolcanic areas [86] may support (without proving) a cause–effect relationship between these findings.

## 6. Increases in Heavy Metals and Thyroid Cancer: A Cause–Effect Relationship?

A cause–effect relationship between chronic exposure to increased metal levels in the volcanic environment and thyroid cancer is difficult to demonstrate via clinical studies in residents of a volcanic area. Therefore, the problem has been approached through in vitro studies in human thyroid cells and in vivo studies in experimental animals.

A major unanswered question is how such small increases in environmental metals, not exceeding what is considered to be the normal range in most cases, can promote the malignant transformation of the human thyroid. A second important question is why the possible carcinogenic effect of increased metal levels in a volcanic area predominantly, though non-exclusively [87], affects the thyroid gland. The identification of the mechanisms involved in these processes is crucial for better understanding the possibility of a cause–effect relationship

### 6.1. Hormesis Effect

Chemical hormesis is a biological phenomenon characterized by a nonlinear response of biological activity (including cell growth and carcinogenesis) to a stimulator [88]. In hormesis, the biological response to increasing amounts of a chemical is biphasic, with biological effects increasing at low concentrations, followed by the inhibition of the effect at higher doses.

Many metals can cause hormetic responses in biological systems. Low levels of potentially toxic heavy metals such as Ag, As, Cd, Hg, and Y can cause stimulatory effects on cellular activities both in vitro [89,90,91,92] and in vivo [93,94,95,96]. The hormetic effects of these metals usually occur in the µM concentration range, and the stimulated increase is less than 100% above the basal level. The response depends on the concentration of the metal studied, the time of exposure, and the target cell examined. In vitro, malignant cells may be unresponsive to metal doses that cause a hormetic response in nontransformed cells [97], and in vivo, this response may be stage dependent, displaying toxicity only in a later developmental period [93,98].

The mechanism of the hormetic effect is unknown. In addition to possible cross-talk among metal ions with similar chemical features, it most likely involves the generation of reactive oxygen species (ROS), which could induce cell damage and activate reactive mechanisms [90,99,100]. Redox reactions can be modulated by the variable availability of transition metals that serve as donors of electrons. Through these mechanisms, low levels of ROS may stimulate the activation of the ERK/MAPK (mitogen-activated protein kinase) signaling pathway and, as a consequence, increase protein synthesis, cell proliferation and differentiation, and resistance to stress conditions [91,92,101]. A potential alternative mechanism is the binding to and inhibition of protein tyrosine phosphatases by metals [102], indirectly increasing the activity of tyrosine kinases.

**Figure 3:**
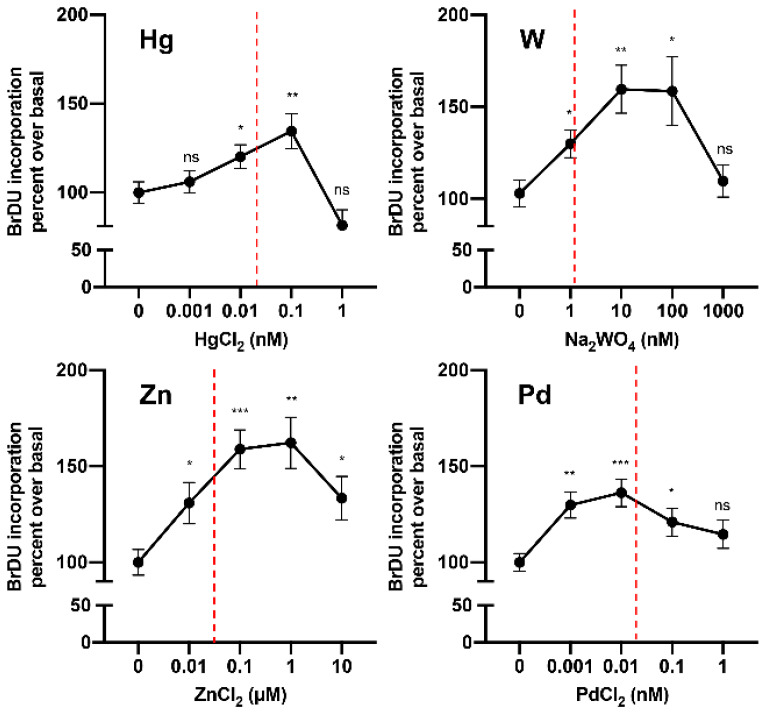
Metals at low concentrations stimulate human thyrosphere proliferation. Proliferation (measured by 5-bromo-2-deoxyuridine (BrDU) incorporation) of human thyrospheres (aggregates of thyroid stem/precursor cells) after chronic exposure to increasing concentrations of Hg, W, Zn, and Pd. Vertical dotted lines indicate the average concentration of each metal in the urine of residents in the volcanic area of Sicily [81].

Until very recently, no data were available on the hormetic effects of metals on thyroid cells. In 2019, in a study of cultured human thyrospheres (spheres containing thyroid stem and precursor cells), it was observed that chronic (days) exposure to low doses of the heavy metal W (applied as sodium tungstate dihydrate) caused a series of biological effects. In vitro, very low concentrations of W stimulated thyrosphere proliferation, as indicated by 5-bromo-2-deoxyuridine (BrdU) incorporation, increased DNA levels, and morphological changes observed under phase-contrast microscopy [103]. These effects were observed at very low W concentrations (nM) within the same range measured in the urine of the residents of the Mt. Etna volcanic area (where thyroid cancer incidence is markedly increased) and disappeared at higher (µM) concentrations. Similar effects on human thyrosphere proliferation were observed in preliminary experiments with Hg and Pd in the nanomolar range and with Zn in the micromolar range (Figure 3). In parallel experiments, no effect of W was observed in differentiated human thyrocytes in primary culture.

A low-level metal-stimulated biological effect in thyrospheres was preceded by the activation of the ERK signaling pathway, while the inhibition of ERK phosphorylation with pertussis toxin inhibited thyrosphere growth [103]. These observations were recently confirmed in cultured thyroid nontransformed cells [96] and suggest a major role of the ERK intracellular pathway in the hormetic effect of tungstate on immature thyroid cell proliferation.

In the same model and at the same concentrations, W inhibited thyroid stem cell differentiation and reduced apoptosis. Moreover, mature thyrocytes derived from thyrospheres chronically exposed to tungstate presented some characteristics typical of transformed cells: they formed more and larger colonies in soft agar and in a clonogenic assay, and they showed a greater migration capacity in a scratch wound-healing assay [103].

**Figure 4:**
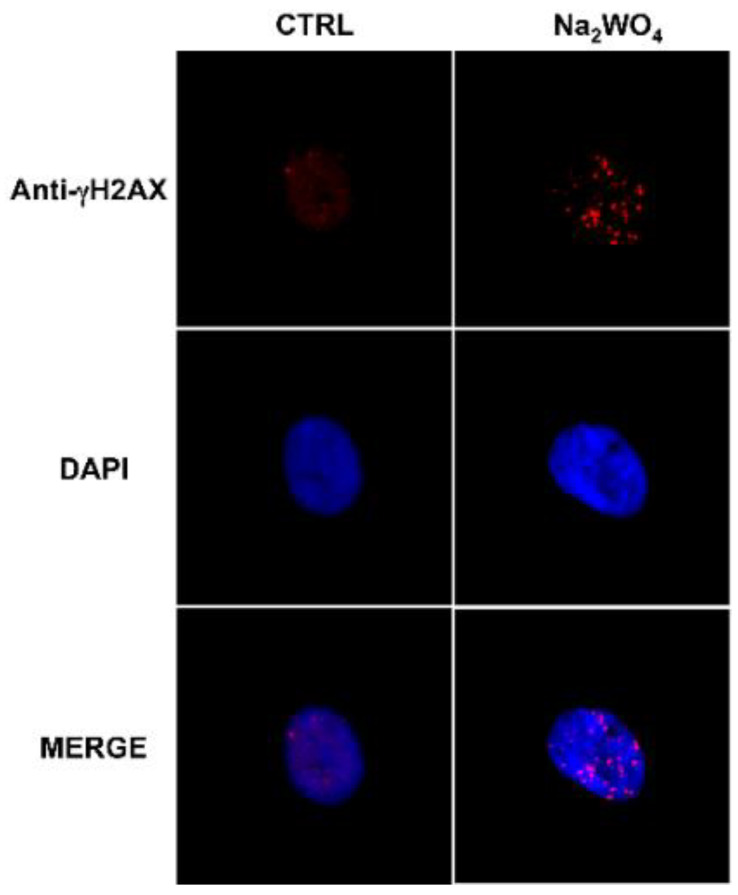
Exposure to a low dose of tungsten affects DNA repair proteins in human thyrospheres. The exposure of human thyrospheres to a low dose of W (Na_2_WO_4_, 30 nM for 90 min) increases the expression of the DNA repair protein γH2AX. γH2AX (red) was detected using a γH2AX antibody followed by an Alexa Fluor-594-conjugated secondary antibody. Nuclei were visualized with DAPI (4′,6-diamidino-2-phenylindole) (blu) [103].

Chronic exposure to low levels of W also altered the genetic profile of thyroid stem/precursor cells and affected DNA repair protein activity [103] (Figure 4).

These in vitro data indicate that chronic exposure to very low concentrations of W, while being harmless to mature thyrocytes, has relevant effects on undifferentiated or partially differentiated thyroid cells. Biphasic hormetic responses to metals have already been described for other types of undifferentiated cells, such as lung embryo fibroblasts and human embryonic kidney cells [100,104]. The novelty of this thyroid model is that exposed progenitor cell abnormalities produce a population of mature thyrocytes with biological characteristics compatible with a preneoplastic state.

The influence of thyroid progenitor cells’ exposure to W on the characteristics of their progeny (mature thyrocytes) is reminiscent of the transgenerational transfer of the hormetic effects of metals reported in plants [105,106] and animals [95]. These findings may have important implications for the estimation of hazard assessments for carcinogenesis and cancer risk in later life. The question of safe metal levels in the environment during the prebirth period and neonatal life, when tissues (including the thyroid) exhibit a high prevalence of stem/precursor cells, is an important issue that deserves further study.

### 6.2. Is Metal Accumulation in the Thyroid a Possible Mechanism Contributing to the Increase in Thyroid Cancer?

As mentioned above, many metals (B, Br, Cd, Co, Cu, Hg, Li, Mn, Mo, Pd, Se, Sn, Tl, U, V, W, and Zn) showed significantly increased levels (*p* < 0.001) in the urine of residents of the volcanic area in Sicily [81]. Therefore, individuals born and living in that area suffer lifelong biocontamination with metals, beginning very early in life.

However, high variability in the biocontamination levels of different metals is observed in individuals living in this area; as a consequence, the concentration ratio of each metal relative to those of the other trace elements is highly variable. Therefore, the effects of these metals on thyroid cells cannot be extrapolated from those observed in vitro with tungsten, as in vivo conditions are much more complex and heterogeneous. Each metal will most likely have different effects on the thyroid when acting separately than when multiple metals are simultaneously present in excess. Metal effects in fact depend not only on the dose but also on the synergistic or antagonistic influences of other metals. The ultimate effect of different, variable combinations of multiple excess heavy metals on the human thyroid is currently unknown.

In this regard, one factor that must be considered is the different capacities of the thyroid to specifically accumulate different metals, which depend not only on environmental exposure but also on metabolic processes such as cell uptake, retention, and clearance. According to the specificity and selectivity of these processes in thyroid cells, different metals may accumulate at higher levels in the thyroid than in other tissues. The selective accumulation of one or more trace elements with a carcinogenic effect could explain the predominant increase in thyroid cancer observed in the presence of environmental heavy metal biocontamination.

Recently, metals were comparatively measured (using inductively coupled plasma mass spectrometry) in normal human thyroid tissue and in sternothyroid muscle and neck subcutaneous fat tissues collected from the same euthyroid individuals. 

As, Br, Cd, Hg, Mn, Se, and Sn showed significantly higher concentrations (*p* < 0.01) in the thyroid than in the other two tissues [107]. Among these elements, As, Cd, and Hg are recognized carcinogens (class 1, IARC). As and Hg (but not Cd, which was under the assay detection limit in all tissues) were also shown to be more concentrated in the thyroid of normal rats relative to the hindlimb muscle and abdominal visceral fat of the same rats.

Whether the relative accumulation of these carcinogenic elements contributes to the very frequent occurrence of thyroid cancer in the volcanic biocontaminated area is unclear. When the metal concentrations measured in the thyroid tissue of the residents of the volcanic biocontaminated area (n = 43) were compared to those found in the residents of the control area (n = 34), 11 out of the 18 examined elements were at slightly higher levels in the thyroid of subjects living in the volcanic area. However, a large overlap of metal levels was found between the two groups, and the differences were not statistically significant. Moreover, in residents of the volcanic area, most metals were increased also in muscle and adipose tissues, suggesting a generalized consequence of increased exposure, rather than a specific thyroid accumulation mechanism. However, this was not the case for As and Hg, which were slightly increased (+16.5% and +25%, respectively) in the thyroid but not in the other examined tissues of residents of the volcanic area. These small differences may be biologically relevant based on the hormetic mechanism.

In conclusion, studies on metal concentrations in the thyroid do not provide evidence of a clear role of the accumulation mechanism in metal-dependent thyroid carcinogenesis, but they are compatible with this possibility.

### 6.3. Metals and Thyroid-Specific Biology

In addition to the possibility that the selective accumulation of carcinogenic metals favors thyroid cancer more than cancers of other tissues, alternative mechanisms may explain why environmental metal pollution predominantly promotes thyroid cancer.

Follicular thyroid cells have peculiar biological properties: their main function is to produce thyroid hormones, which requires iodine. Iodine is a very reactive element and a strong oxidizing agent. Thyroid cells take up iodine via a specific sodium/iodine symporter (NIS) in the form of iodide anion (I^-^), which is readily oxidized by the thyroid-specific enzyme thyroperoxidase (TPO). Therefore, thyroid cells are constantly exposed to free radicals produced by the continuous generation of hydrogen peroxide (H_2_O_2_) by the NADPH (nicotinamide adenine dinucleotide phosphate hydrogen) oxidase Duox. H_2_O_2_ is necessary for I^-^ oxidation to produce derivatives such as hypoiodite, hypoiodous acid, and iodate [108]. This intracellular chemistry produces high oxidative stress, which may in turn favor spontaneous mutagenesis. In fact, in an experimental model, the mutation rate in the thyroid is 8–10 times higher than that in the liver [109]; under these conditions, the additional free radicals produced by increased metal exposure can more easily cause DNA damage and cell transformation.

Other peculiar thyroid cell characteristics may be involved in increased thyroid sensitivity to metals present in excess, as indicated by the complete inhibition of the enzyme xanthine oxidase by tungsten in the rat thyroid [110].

The possible causal relationship between metals and thyroid cancer is supported by the effect of the heavy metal copper on BRAF^V600E^ mutation-driven carcinogenesis. The papillary histotype is the most frequent (over 80%) thyroid cancer histotype and is due to the oncogenic mutation of BRAF in most cases (over 50%). Copper chelation inhibits MEK 1/2 kinase activity, and reduced MAPK signaling inhibits BRAF^V600E^-driven melanoma growth [111]. The same effect was observed in BRAF^V600E^-positive human PTC cells and in a genetically engineered mouse PTC model [112], indicating the possibility that increased copper may favor the occurrence of thyroid cancer with papillary histotype.

### 6.4. In Vivo Data in Experimental Animals

A well-accepted system for evaluating the cause–effect relationship between suspected carcinogens and the actual induction or promotion of cancer is to use experimental animal models. If the feeding of animals (mainly rodents) with a diet containing elevated concentrations of metals results in the appearance of signs of malignant transformation, it is assumed to indicate the carcinogenicity of the tested compound.

However, these models have important limitations. Animals show differences in sensitivity to metals relative to humans as a consequence of differences in absorbance, tissue accumulation, and clearance, metabolism, and excretion.

Moreover, species specificity has been observed; for example, As compounds may have carcinogenic effects in the mouse thyroid but not in the rat thyroid [113].

Many studies on metal carcinogenicity have been carried out in animals in a variety of tissues, but only a few of these studies focused on the thyroid, and most of them mainly concerned thyroid function, rather than carcinogenesis.

In addition, these animal experiments presented major problems regarding metal compound dosage and time of exposure, as in most cases, high doses (mM range) and a short exposure time (weeks) were used. When low exposure was examined, As compounds have been shown to disrupt T_4_ homeostasis and influence related gene transcription after 8 weeks of exposure, but no morphological modifications were described [114]. Moreover, male rats exposed to bromine (KBrO_3_) in quantities calculated to be within the high range expected in the environment exhibited morphological goiter-like changes in the thyroid after 66 days [115] and increases in the number of mitoses and vascularization after 133 days [116]. After longer exposure (animals exposed to similar KBrO_3_ concentrations for up to two years), carcinogenic effects were observed in the thyroid and kidneys of rats but only in the kidneys of mice [117].

These data reflect an experimental condition very different from that observed in polluted volcanic areas, where not only is there a mixture of many metals present, but metal concentrations in the environment are also in the high–normal range and exposure occurs throughout an individual’s life, including the prenatal stage [81].

To evaluate conditions better mimicking chronic exposure to low-dose metal pollution, a study was recently carried out in a well-established in vivo model of thyroid tumorigenesis [118]. Female rats were supplied with a goitrogenic diet, and B, Cd, and Mo were added to their drinking water at concentrations twice as high those measured in the urine of the residents of the volcanic area in Sicily. The thyroids of treated animals examined after 5 and 10 months of exposure exhibited progressive increases in follicular dyshomogeneity, nuclear pseudoinclusions, and papillary structures compared to the thyroids of the control rats (also hypothyroid under the goitrogenic diet) [119]. Papillary structures associated with nuclear aberrations are considered preneoplastic features of the thyroid. An increase in such structures indicates that chronic exposure to B, Cd, and Mo, even at low levels, accelerates the neoplastic characteristics of the thyroid induced by the goitrogenic diet. The study confirms that under conditions causing a predisposition to neoplastic transformation, chronic exposure to slightly increased B, Cd, and Mo levels may favor thyroid cancer initiation acting as tumor-promoting agents, rather than as true carcinogens. However, this model is quite different from the in vivo conditions of the residents of the volcanic area and is inadequate for documenting the carcinogenic potential of lifelong exposure to multiple metals.

## 7. Concluding Remarks

In recent decades, metal pollution of the environment has increased worldwide [120], highlighting the question of its possible deleterious effects on human health. The thyroid is a histologically and functionally complex gland. Its major functions of iodine uptake and incorporation into tyrosine residues to produce thyroid hormones require specific oxidoreduction processes that can make the thyroid more vulnerable to toxic heavy metals.

The health concern related to the anthropogenic pollution of the environment with metals is a serious issue and a worsening problem. As a result of its biochemistry and biology, the thyroid may act as a sensitive and precocious indicator of the possible health damage caused by environmental heavy metal pollution.

At present, we lack solid data on the carcinogenic effect of increased environmental metal levels on thyroid cancer initiation and progression. The available evidence is indirect, circumstantial, and incomplete.

More in general, our understanding of the metal pollution and its consequences on human health is totally inadequate considering the complexity and variability of the interactions of different cells with different metals, different metal doses and lengths of exposure, the different speciation of each metal, and the competing or potentiating effects metals on each others’ activities.

Further rigorous and innovative studies on this issue aimed at identifying the mechanisms of action and the biological effects triggered by chronic exposure to slightly increased metal concentrations are warranted. More specifically, a better understanding of the additive, synergistic, or antagonistic effects of different metals with other organic and inorganic compounds in the environment is required. An improved understanding of these issues would greatly contribute to our comprehension of the relationship between environmental metal pollution and increased health damage, including thyroid cancer.

## Figures and Tables

**Table 1:** Thyroid cancer in Sicily: age-standardized incidence rates for the world population (ASRw) in the volcanic and the control areas and the papillary/follicular histotypes ratio. Data from [35].

Environment	Inhabitants (millions)	Thyroid Cancer Incidence (ASRw)		Papillary/Follicular Ratio
		F	M	
Volcanic area (Catania province)	1116	31.7	6.4	25.9
Control area (all Sicily without Catania)	3853	14.1	3.0	9.8
